# Inhibition of SGLT2 reduces blood pressure in the early phase of salt‐sensitive hypertension in male Dahl‐SS rats independently of changes in renal inflammation

**DOI:** 10.14814/phy2.70998

**Published:** 2026-07-03

**Authors:** Rawan N. Almutlaq, Yotesawee Srisomboon, Sridhatri Guntipally, Andrew N. Hakeem, Amanda C. Veiga, Jaryd Ross, Babatunde S. Anidu, Alex Dayton, Scott M. O'Grady, Louise C. Evans

**Affiliations:** ^1^ Department of Integrative Biology and Physiology University of Minnesota Minneapolis Minnesota USA; ^2^ Department of Animal Science University of Minnesota Minneapolis Minnesota USA; ^3^ Department of Surgery, Division of Autonomic Neuromodulation University of Minnesota Minneapolis Minnesota USA; ^4^ Department of Medicine, Division of Nephrology and Hypertension University of Minnesota Minneapolis Minnesota USA; ^5^ Present address: Department of Physiology, Escola Paulista de Medicina Universidade Federal de Sao Paulo Sao Paulo Brazil

**Keywords:** albumin, inflammation, salt‐sensitivity

## Abstract

Salt‐sensitive hypertension is a progressive condition characterized by albuminuria, renal injury, and inflammation. The initiating mechanisms remain unclear. We hypothesized that early in salt‐sensitive hypertension, the proximal tubule is exposed to excess albumin, thereby triggering cytokine release and renal inflammation. Blood pressure, renal injury, and inflammation were assessed in Dahl salt‐sensitive (SS) rats fed a 4.0% NaCl high‐salt (HS) diet for 7 days. Using a proximal tubule cell line, we tested whether albumin exposure triggers epithelial cytokine release, and if this is reduced by dapagliflozin. Lastly, we examined whether dapagliflozin modified the response to 7 days HS in SS rats. Urinary albumin and CCL2 were higher in SS rats fed HS than those fed control salt, prior to differences in blood pressure between the groups. After 7 days, renal macrophage accumulation was higher in HS fed SS rats and correlated positively with albuminuria. Albumin induced CCL2 release from cultured proximal tubule cells; this was prevented by dapagliflozin cotreatment. In SS rats, dapagliflozin blunted the development of salt‐induced hypertension but didn't reduce renal macrophage accumulation. Albuminuria is a primary event in SS hypertension and is correlated with renal macrophage accumulation. Inhibition of SGLT2 lowers blood pressure but does not reduce renal inflammation.

## 1 INTRODUCTION

Hypertension is the leading modifiable risk factor for cardiovascular disease worldwide (Kearney et al., 2005). Approximately 50% of patients diagnosed with essential hypertension have salt sensitivity, a condition in which blood pressure increases in response to elevated salt intake (Weinberger et al., 2001). Salt sensitivity is strongly linked to an increased risk of kidney damage (Elijovich et al., 2016; Weinberger, 1996). Despite extensive research, the precise mechanisms underlying salt‐sensitive hypertension remain largely unknown. Determining the underlying factors is critical for understanding the pathogenesis and the most effective strategies for managing the disease.

The Dahl salt‐sensitive (SS) rat is a widely used and well‐characterized model that closely mimics salt‐sensitivity in humans, exhibiting progressive hypertension, proteinuria, and renal injury when challenged with a high‐salt (HS) diet (Cowley Jr. et al., 2013; Kato, 1999; Mattson, 2014; Rapp, 2000). Studies have shown that salt‐sensitive hypertension in SS rats develops in two phases (Cowley Jr. et al., 2013; De Miguel et al., 2010; Evans et al., 2025; Mattson, 2014). The primary phase occurs during the first week of a high‐salt diet and is characterized by increased urinary albumin and reduced medullary blood flow. The secondary phase occurs during the second 2 weeks of high‐salt and is characterized by reduced glomerular filtration rate (GFR) and increased infiltration of renal immune cells. Servo‐control studies have shown that increased renal perfusion pressure amplifies renal inflammation in SS rats; however, the initiating factors within the kidney remain elusive (Cowley Jr. et al., 2013; Evans et al., 2017).

While vascular and glomerular mechanisms of injury have been widely studied, increasing attention has focused on the proximal tubule as a key initiator of renal inflammation (Tang et al., 2003; Wang et al., 1997; Zhou et al., 2021). As a particularly metabolically active segment of the nephron, the proximal tubule is highly susceptible to stressors such as albumin overload, which can stimulate the production of proinflammatory chemokines and cytokines. Among these, CCL2 (C‐C motif chemokine ligand 2), also known as monocyte chemoattractant protein‐1 (MCP‐1), is a potent chemoattractant that recruits monocytes and macrophages to sites of tissue injury and plays a key role in renal macrophage infiltration in salt‐sensitive hypertension (Liu et al., 2015; Wagner et al., 2016).

The sodium‐glucose cotransporter‐2 (SGLT2), primarily expressed in the proximal tubule, plays a crucial role in glucose and sodium reabsorption (Perry & Shulman, 2020). Clinical trials have demonstrated that SGLT2 inhibitors not only lower blood glucose but also reduce cardiovascular events, lower blood pressure, and slow progression to end‐stage kidney disease in patients with type 2 diabetes mellitus (Baker et al., 2014; Gonzalez et al., 2020; Neal et al., 2017; Perkovic et al., 2019; The E‐KCG et al., 2023). In addition, SGLT2 inhibitors have demonstrated renoprotective properties, including reductions in albuminuria in CKD management (Heerspink et al., 2024; Wada et al., 2022). In preclinical models, SGLT2 inhibition attenuates salt‐induced hypertension in SS rats (Kravtsova et al., 2022), however, the impact of SGLT2 inhibition on early renal inflammation and immune cell recruitment in salt‐sensitive hypertension has not been examined. We hypothesized that exposure of the proximal tubule to excess albumin triggers cytokine release from the epithelium which may be involved in the initiation of renal inflammation in salt‐sensitive hypertension. Further, we tested whether reducing metabolic workload, via the administration of dapagliflozin, reduced the pathological effects of albumin.

In the current study, we demonstrated that albuminuria is an early event in the development of salt‐sensitive hypertension in SS rats. Urinary albumin was higher in SS rats fed HS than those fed control‐salt (0.4% NaCl, CS) from day 1 of the dietary challenge; this was despite no differences in mean arterial blood pressure between the groups at that time. Albuminuria was paralleled with increased urinary CCL2 and correlated with renal accumulation of macrophages but not T‐cells. Using an immortalized proximal tubule cell line, we demonstrated that apical albumin induced CCL2 release from both the apical and basolateral membranes, and this effect was prevented by dapagliflozin pretreatment, which inhibits SGLT2. Administration of dapagliflozin to SS rats reduced the blood pressure response to 7 days of high salt; but, contrary to our hypothesis, it did not mitigate urinary albumin or renal inflammation.

In summary, these studies highlight albuminuria as an early event in the development of salt‐sensitive hypertension in SS rats. Within the first 24 h of the dietary challenge, urinary albumin was more than two‐fold higher in the HS fed SS rats than in those fed CS. This was despite comparable blood pressures between the groups at this time point. Albuminuria was correlated with renal macrophage accumulation. Contrary to our hypothesis, the early anti‐hypertensive effects of SGLT2 inhibition occur independently of reductions in albuminuria or renal immune cell accumulation.

## 2 MATERIALS AND METHODS

### 2.1 Experimental animals

Male SS rats were obtained from colonies maintained at the Medical College of Wisconsin. From weaning, all rats were maintained on a purified CS rodent diet containing 0.4% NaCl (AIN‐76A, Cat# 113755, Dyets, Bethlehem, PA) and had ad libitum access to water. When specified, rats were challenged with a HS diet containing 4.0% NaCl (AIN‐76, Cat# 113756, Dyets, Bethlehem, PA). Three groups of rats were assessed: (1) blood pressure analysis by telemetry, (2) urinary analysis, and (3) SGLT2 inhibition.

All experimental procedures were approved by the Institutional Animal Care and Use Committee (IACUC) at the University of Minnesota and adhered to the NIH Guide for the Care and Use of Laboratory Animals. Rats were individually housed in temperature‐controlled rooms on a 12‐h light/dark cycle (7:00 am to 7:00 pm).

### 2.2 Surgical preparation for the assessment of blood pressure by telemetry

As previously described (Dayton et al., 2024), 9–11‐week‐old rats were surgically prepared for the assessment of blood pressure by radiotelemetry devices. Under isoflurane anesthesia (5% induction, 2%–3% maintenance), the telemeter catheter (Model HD‐S10, Data Sciences International, MN) was inserted into the femoral artery for continuous blood pressure assessment. After a recovery period of 6 days, blood pressure was recorded over 3 baseline days, during which rats were fed CS, followed by 7 days of HS challenge and euthanized on day 8.

### 2.3 Metabolic cage study and urinary analysis

A separate subset of rats was housed in metabolic cages for daily 24‐h urine collections. Rats were acclimatized to the cages for 2 days before daily urine collections began. Urine collections were made over 3 days of baseline CS and 7 days of HS challenge. Urine samples were collected every 24 h, aliquoted, and stored at −80°C for further analysis.

Urinary protein was quantified using the Bradford Protein Assay Kit (Cat# 5000205, Bio‐Rad). Urinary albumin, kidney injury molecule‐1 (KIM‐1), and CCL2 were measured by ELISA per the manufacturer's instructions (Albumin: Cat# OKIA00131, Aviva Systems Biology; KIM‐1: Cat# RKM100, R&D Systems; CCL2: Cat# RAB0057‐1KT, Sigma‐Aldrich). Electrolyte levels (Na^+^, K^+^, Cl^−^) were measured using a SmartLyte Plus Analyzer (Diamond Diagnostics, Holliston, MA), and osmolality was determined using a freeze‐point depression osmometer (Micro‐Osmometer, Advanced Instruments, Norwood, MA). Cytokine and chemokine levels were measured in urine using a 9plex assay, with the Luminex Discovery Assay (Cat# LXSAHM, R&D Systems). Concentrations were calculated from standard curves generated for each cytokine (IL‐1α, CXCL10, EGF, IL‐13, IL‐4, Eotaxin, IL‐12p70, GRO/KC, VEGF, CX3CL1, G‐CSF, GM‐CSF, Leptin, MIP‐1α, IL‐1β, IL‐2, IL‐6, IL‐10, IFN‐γ, IL‐5, IL‐17A, IL‐18, LIX, MIP‐2, TNF‐α, RANTES). Only cytokines with values consistently within the assay's detection range were included in quantitative analyses. Cytokines with values below the lower range of the standard curve were excluded from statistical analysis. Urinary cytokines were normalized to creatinine, which was measured using a colorimetric assay (Cat# ab204537, Abcam). All assay plates were run according to the manufacturer's protocol. Assay results were analyzed using Belysa Analysis software, version 1.2.2. All ELISA‐based assays, including urinary protein (Bradford), albumin, KIM‐1, CCL2, and the multiplex cytokine panel, were performed in technical duplicate for each sample. Urinary electrolytes and osmolality were measured in singlicate.

### 2.4 SGLT2 inhibition

A third subset of rats was randomly assigned to receive either the SGLT2 inhibitor dapagliflozin in 1 g of Nutella (DAPA; 2 mg/kg/day, Cat# SML2758, Sigma‐Aldrich, St. Louis, MO) or vehicle (Nutella) by oral administration. DAPA treatment began on day −2 when rats were fed CS.

### 2.5 Tissue collection

Rats were deeply anesthetized with isoflurane (5% induction, 2%–3% maintenance) and aortic blood collected. This was followed by perfusion of both kidneys with normal saline and then euthanization by exsanguination. The left kidney was fixed in 10% neutral buffered formalin for 48 h, and transferred to 70% ethanol for storage prior to histological analysis. The right kidney was dissected into outer medulla and cortex, snap frozen in liquid nitrogen, and stored at −80°C. In the subset of rats used in the DAPA study, the upper pole of the left kidney was isolated for flow‐cytometry analysis.

### 2.6 Flow cytometry and immune cell profiling

As previously described (Dayton et al., 2024; Evans et al., 2017), immune cells were isolated from kidney tissue. In brief, tissues were minced and digested in RPMI‐1640 containing collagenase type IV (Cat#LS004210, Worthington) and DNase I (Cat# DN25, Sigma‐Aldrich) in gentleMACS C tubes (Cat# 130–093‐237, Miltenyi Biotec), then filtered through 100 μm, 70 μm, and 40 μm cell strainers (Cat# 352360, 352,350, 352,340, Corning). Mononuclear cells were isolated using a Percoll gradient (Cat# P1644, Sigma‐Aldrich; 30% over 70%). Cells were stained in PBS + 0.5% BSA with the following antibodies: anti‐rat CD45‐PE‐Cy7 (Cat# 202213, BioLegend; Clone OX‐1), anti‐rat CD3‐PerCP‐eFluor 710 (Cat# 46–0030, eBioscience; Clone G4.18), anti‐rat CD8a‐FITC (Cat# 201703, BioLegend; Clone OX‐8), anti‐rat CD4‐APC‐Cy7 (Cat# 201517, BioLegend; Clone W3/25), anti‐rat CD45R‐PE (Cat# 554881, BD Biosciences; Clone HIS24), and anti‐rat CD11b/c‐eFluor 660 (Cat# 50–0110‐82, eBioscience; Clone OX‐42). Samples were acquired on a BD LSR II flow cytometer and analyzed in FlowJo (Ashland, OR) in a blinded manner.

### 2.7 Histological analysis

Kidneys were embedded in paraffin, cut into 4 μm sections, and stained with Masson's Trichrome. Glomerular injury was assessed in a blinded fashion as previously described (De Miguel et al., 2010; Semenikhina et al., 2024). A total of 65–70 cortical glomeruli per kidney were scored on a scale of 0–4, with 0 indicating the healthiest and 4 indicating the most severely damaged. Protein cast was quantified using QuPath (version 0.5.1) as the percentage of tubular protein cast‐positive area. Fibrosis was quantified using standardized color thresholding in ImageJ and expressed as a percentage of the collagen‐positive area within the kidney section. Additional sections were stained with an anti‐human, rabbit polyclonal CD3+ antibody to localize T cells (Cat# A0452, Dako/Agilent, 1:200 dilution) and with anti‐human, rabbit polyclonal Iba1 (ionized calcium‐binding adaptor molecule 1) antibody to localize macrophages (Cat# CP290B, Biocare, 1:600 dilution) using immunohistochemistry. Negative controls lacked primary antibodies. Images were captured at 10× magnification (Keyence BZ‐X800) and quantified in ImageJ and QuPath. To assess renal inflammation in the cortex and outer medulla, regions of interest were traced and threshold analysis performed in each area separately.

### 2.8 In vitro studies

Human renal proximal tubule epithelial cells (RPTEC/TERT1, Cat# CRL‐4031, ATCC) were grown on Transwell inserts (insert size: 0.4 μm pore size, Cat# 3450, Corning) to establish polarized monolayers. Transepithelial electrical resistance (TEER) was measured to verify monolayer integrity and confirm tight junction formation prior to experimentation. All cells were grown in DMEM: F12 medium (ATCC Cat# 30–2006) with hTERT RPTEC growth kit (ATCC Cat# ACS‐4007).

Four experimental conditions were tested for 24 h and 48 h (*n* = 6 per group): (1) Control media alone, (2) Human serum albumin (HSA, Cat# A3782, Sigma‐Aldrich, 10 mg/mL (Zoja et al., 1998)), (3) Dapagliflozin (DAPA, Cat# SML2804, Sigma‐Aldrich, 15 ng/mL (Eleftheriadis et al., 2022)), and (4) HSA + DAPA. Apical and basolateral media were collected separately and analyzed for CCL2 (Cat# DCP00, R&D Systems) and KIM‐1 (Cat# DSKM100, R&D Systems) following the manufacturer's instructions.

### 2.9 Statistical analysis

Data are presented as mean ± SD. Repeated‐measures outcomes were analyzed using linear mixed‐effects models (LMM), with fixed effects for diet, treatment, and day as appropriate. Tukey's post hoc test was used for multiple comparisons. Single time point comparison between two groups was performed using unpaired Student's *t*‐tests. Comparisons involving more than two groups at a single time point were analyzed using one‐way ANOVA with Sidak post hoc comparison. The specific statistical test used is indicated in the figure legend for each figure. Analyses were performed using GraphPad Prism (version 10 for *t*‐tests and ANOVA‐based analysis) and R for mixed effects modeling. A *p*‐value < 0.05 was considered statistically significant.

## 3 RESULTS

### 3.1 Early blood pressure responses to a high salt diet in male Dahl SS rats

**FIGURE 1:**
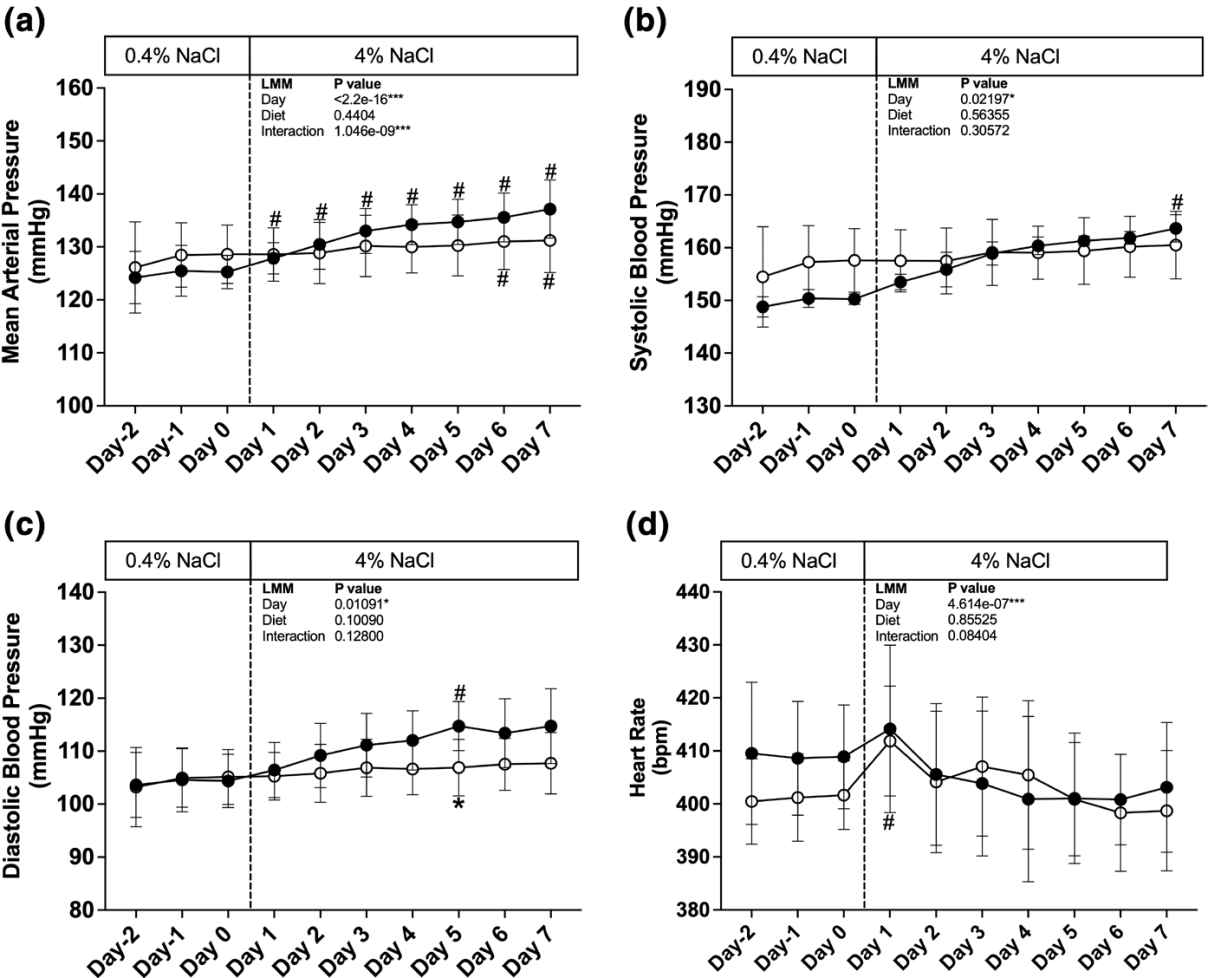
Blood pressure and heart rate responses to high salt diet in male Dahl SS rats. Telemetry measurements of arterial pressure and heart rate in male Dahl salt‐sensitive rats during baseline control salt diet (CS, 0.4% NaCl, white circles) and following transition to a high‐salt diet (HS, 4% NaCl, black circles). (a) Mean arterial pressure (MAP), (b) systolic blood pressure (SBP), (c) diastolic blood pressure (DBP), and (d) heart rate (HR) are shown over time. The dashed vertical line denotes the switch from the CS diet to the HS diet (day 0). Data are presented as mean ± SD, *n* = 6/group. Statistical analysis was performed using a linear mixed‐effects model with day and diet as fixed effects and animal as a random effect, corresponding *p* values for main effects and interactions are shown within each panel. Post hoc comparisons were made using a Tukey's test. *p < 0.05 CS versus HS. #*p* < 0.05 versus baseline.

To determine the early blood pressure response to HS intake, mean arterial pressure (MAP), systolic blood pressure (SBP), diastolic blood pressure (DBP), and heart rate (HR) were recorded by telemetry in conscious male SS rats. Over the 7 days of the dietary challenge, MAP gradually increased relative to baseline in both HS and CS fed SS rats. Consequently, over the 7 days of salt challenge, there was no difference in MAP between the groups (Figure 1a). Comparable results were obtained for SBP (Figure 1b) and HR (Figure 1d), which did not differ between SS rats fed CS and HS. DBP was transiently higher in HS fed SS rats on day 5, but this difference between groups did not persist on days 6 and 7 (Figure 1c).

### 3.2 HS diet rapidly induces increased urinary output, proteinuria, albuminuria, tubular injury markers, and inflammatory cytokines in male Dahl SS rats

**FIGURE 2:**
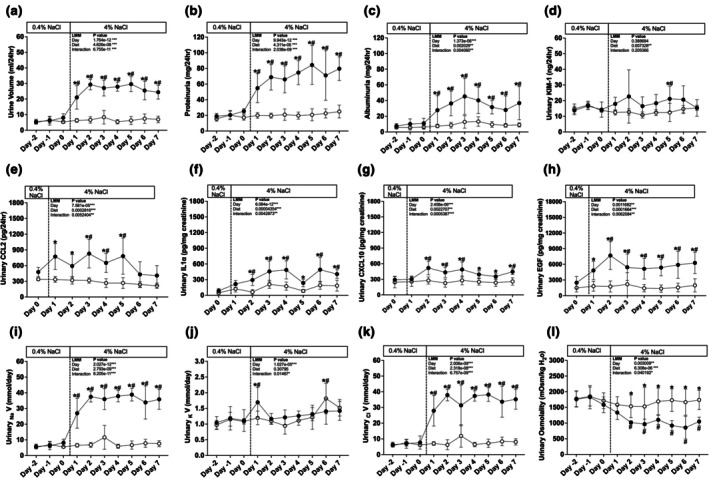
High‐salt intake induces early proteinuria, tubular injury markers, and excretion of inflammatory chemokines in male Dahl salt‐sensitive rats. Effects of high salt intake on (a) urine volume, (b) total proteinuria, (c) albuminuria, and (d) urinary KIM‐1 excretion measured over the time course. (e–h) Urinary excretion of inflammatory mediators, including (e) CCL2, (f) IL‐1α, (g) CXCL10, and (h) EGF. (i–k) Urinary electrolyte excretion of (i) sodium, (j) potassium, and (k) chloride. (l) Urinary osmolality. Rats were maintained on a control salt diet, followed by a transition to a high‐salt diet; the dashed vertical line denotes the initiation of HS intake (day 0). Rats maintained on a control salt diet throughout are represented by white circles. Rats switched to a high salt diet are represented by black circles. Data are presented as mean ± SD; *n* = 6/group. Statistical analysis was performed using a linear mixed‐effects model with day and diet as fixed effects and animal as a random effect; corresponding *p* values for main effects and interactions are shown within each panel. Tukey's post hoc test was used for multiple comparisons. *p < 0.05 CS versus HS. #*p* < 0.05 versus baseline.

To determine the early renal response to HS intake, urinary volume, protein, albumin, tubular injury markers, and inflammatory mediators were measured daily during the transition from CS to HS diet (Figure 2). HS intake resulted in a rapid and sustained increase in urine volume within 24 h of the diet transition, which was higher in SS rats fed HS than in those fed CS from day 1 of the dietary challenge (Figure 2a). Comparable results were obtained for urinary protein excretion and urinary albumin excretion, both of which were higher in HS fed SS rats than CS fed SS rats from day 1 of the dietary challenge. In contrast, urinary KIM‐1, a marker of proximal tubular injury, was higher in HS fed SS rats only on day 5 (Figure 2d). Of the 27 urinary cytokines assessed, 9 were detectable at quantifiable levels; however, only four demonstrated a significant main effect of diet in the linear mixed model (Figure 2e–h). CCL2 (Figure 2e), IL‐1α (Figure 2f), CXCL10 (Figure 2g), and EGF (Figure 2h) were higher in HS fed rats, and post hoc comparisons revealed that this was evident from either day 1 or 2 of the dietary challenge. All other detectable cytokines, which did not have a significant main effect of diet, are shown in Table 1. Urinary sodium and chloride excretion were significantly higher in HS fed SS rats (Figure 2i,k), whereas urinary potassium levels were comparable between groups (Figure 2j). Urine osmolality was significantly lower in HS fed rats (Figure 2l).

**TABLE 1:** Time course of urinary cytokine concentrations during the early phase of salt‐sensitive hypertension.

Cytokines	Day 0	Day 1	Day 2	Day 3	Day 4	Day 5	Day 6	Day 7
**Control salt group**								
IL‐4	21 ± 19	25 ± 21	22 ± 22	30 ± 22	18 ± 18	17 ± 15	12 ± 19	15 ± 24
IL‐13	6 ± 4	7 ± 4	7 ± 4	7 ± 6	6 ± 4	5 ± 4	2 ± 4	6 ± 4
IL‐12P70	17 ± 13	17 ± 12	19 ± 11	19 ± 11	16 ± 15	16 ± 12	11 ± 12	13 ± 13
VEGF	419 ± 137	506 ± 184	509 ± 69	425 ± 101	495 ± 103	442 ± 91	467 ± 169	456 ± 106
CX3CL1	155 ± 68	183 ± 83	194 ± 52	158 ± 51	157 ± 59	138 ± 51	147 ± 67	174 ± 83
**High salt group**								
IL‐4	43 ± 47	38 ± 34	96 ± 79	33 ± 51	21 ± 50	43 ± 51	59 ± 95	0.0 ± 0.0
IL‐13	11 ± 10	7 ± 6	21 ± 18	4 ± 7	0.4 ± 1	11 ± 13	16 ± 23	1.3 ± 2
IL‐12P70	30 ± 28	29 ± 23	59 ± 44	15 ± 8	38 ± 46	24 ± 28	27 ± 33	18 ± 24
VEGF	511 ± 140	389 ± 79	371 ± 67	322 ± 60^a^	360 ± 98^a^	326 ± 66^a^	349 ± 117^a^	406 ± 76
CX3CL1	227 ± 86	168 ± 25	230 ± 61	190 ± 60	211 ± 68	198 ± 45	232 ± 102	247 ± 59

*Note*: Urine samples were collected daily from rats maintained on control‐salt (CS) or HS diets (*n* = 6/group). Cytokines were quantified using a multiplex assay, expressed relative to urinary creatinine (pg/mg) and are presented as mean ± SD. Statistical analysis was performed using linear mixed‐effects models with diet and day as fixed effects and animal as a random effect. Cytokines shown in the table were expressed at detectable level but did not have a significant main effect for diet. Tukey's post hoc tests were performed on those with a significant interaction effect.

^a^ *p* < 0.05 versus baseline.

Collectively, these results demonstrate that male SS rats exhibit an immediate and pronounced renal response to HS intake, characterized by proteinuria, albuminuria, and increased urinary levels of CCL2, IL‐1α, CXCL10, and EGF. Given the early development of albuminuria and urinary cytokines, we next examined whether these changes were associated with structural injury and renal immune cell accumulation.

### 3.3 HS diet induces tubular protein cast formation and early macrophage accumulation, which correlates with albuminuria in SS rats

**FIGURE 3:**
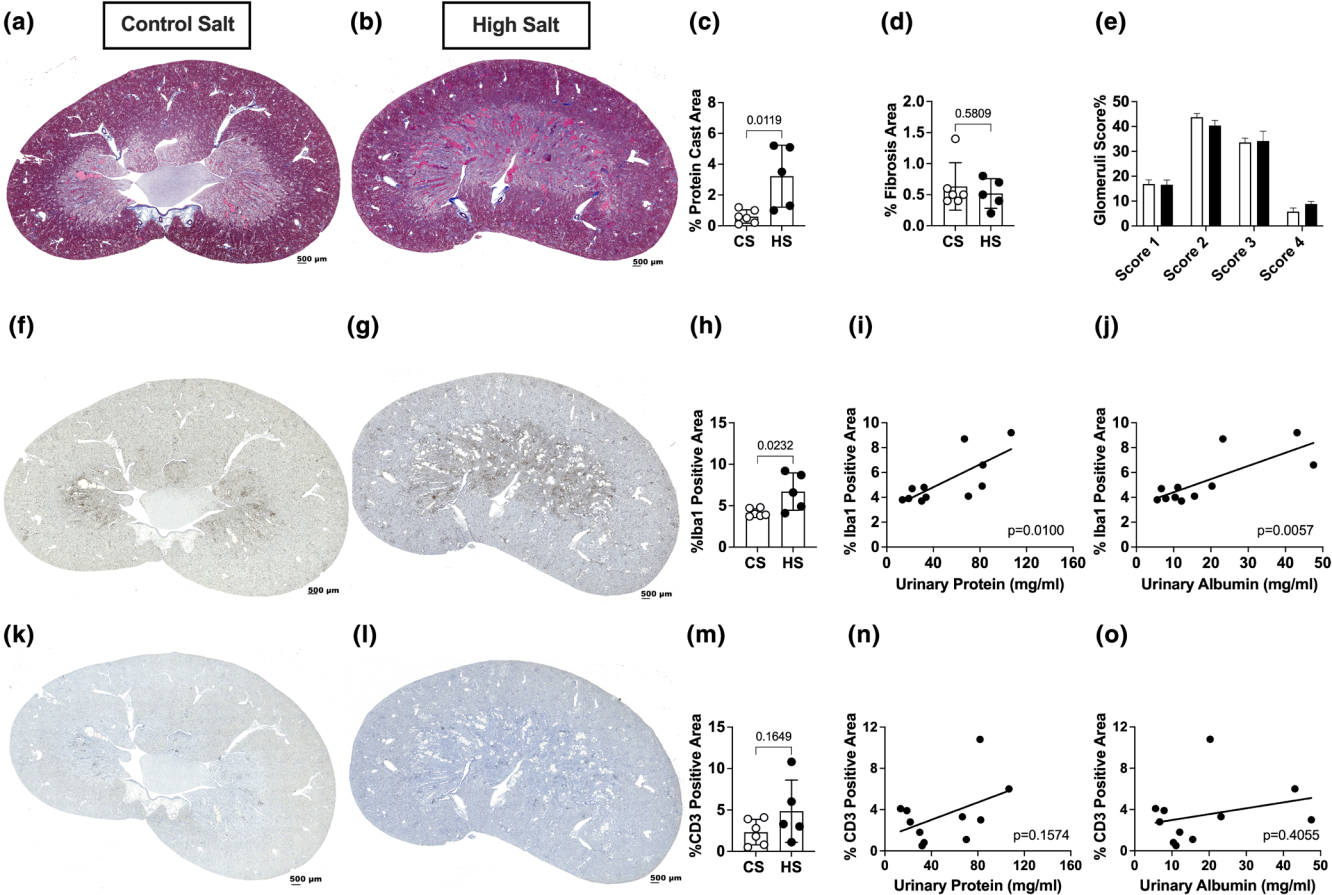
High‐salt intake induces early tubular protein cast formation and renal macrophage accumulation without overt fibrosis in male Dahl salt‐sensitive rats. (a, b) Representative whole‐kidney sections stained with Masson's trichrome from CS and HS rats, respectively. (c) Quantification of tubular protein cast area expressed as a percentage of the total renal area. (d) Quantification of renal fibrosis expressed as a percentage of the total renal area. (e) Distribution of glomerular injury scores in CS and HS rats. (f, g) Representative whole‐kidney immunohistochemical staining for Iba1 (macrophages) in CS and HS rats, respectively. (h) Quantification of total Iba1‐positive area. (i) Correlation between urinary protein excretion and Iba1‐positive area. (j) Correlation between urinary albumin excretion and Iba1. (k, l) Representative whole‐kidney immunohistochemical staining for CD3‐positive T cells in CS and HS rats, respectively. (m) Quantification of total CD3‐positive area. (n) Correlation between urinary protein excretion and CD3‐positive area. (o) Correlation between urinary albumin excretion and CD3. Data are presented as mean ± SD; *n* = 5,6 rats per group. Statistical comparisons were performed using unpaired Student's *T*‐tests or linear regression for correlation analyses, as appropriate. A *p*‐value of < 0.05 was considered statistically significant.

To determine whether early HS intake is associated with structural renal injury and immune cell accumulation, kidney histology and immunostaining were performed following 7 days of HS or CS feeding (Figure 3). Representative Masson's trichrome–stained kidney sections from both the CS and HS groups are shown in Figure 3a,b. Quantitative analysis revealed a significant increase in tubular protein casts (expressed as a percentage of the entire kidney section area) in HS fed rats compared with CS controls (Figure 3c, *p* = 0.0119). In contrast, total fibrotic area did not differ between groups (Figure 3d, *p* = 0.5809). Quantitative glomerular injury scoring found no shift toward severe glomerular injury in the HS fed SS rats (Figure 3e).

To assess renal immune cell accumulation, kidneys were stained for the macrophage marker Iba1 and the T‐cell marker CD3. HS intake resulted in a significant increase in total Iba1^+^ area compared with CS rats (Figure 3h, *p* = 0.0232), indicating early macrophage accumulation within the renal parenchyma. In contrast, the CD3^+^ T‐cell area was not significantly altered during HS intake (Figure 3m, *p* = 0.1649), suggesting that macrophages represent the predominant immune population recruited during the early phase of HS‐induced renal injury.

To determine whether immune cell accumulation was associated with the degree of proteinuria and albuminuria, correlation analyses were performed. Urinary protein levels correlated positively with Iba1^+^ macrophage area (Figure 3i, *p* = 0.0100), and similarly, a positive association was observed between urinary albumin and Iba1^+^ area (Figure 3j, *p* = 0.0057). In contrast, no significant correlations were observed between urinary protein or albumin levels and CD3^+^ T‐cell area (Figure 3n,o, *p* = 0.1574, *p* = 0.4055, respectively). Together, these findings demonstrate that early HS intake in male SS rats induces tubular protein cast formation and macrophage accumulation, occurring in the absence of fibrosis or T‐cell infiltration. Given the strong association between albuminuria and macrophage accumulation in vivo, and the role of CCL2 in macrophage recruitment (Deshmane et al., 2009) and SS hypertension (Alsheikh et al., 2020), we next examined whether albumin directly stimulates CCL2 release from proximal tubular epithelial cells.

### 3.4 Albumin exposure stimulates tubular KIM‐1 and CCL2 release in proximal tubular epithelial cells

**FIGURE 4:**
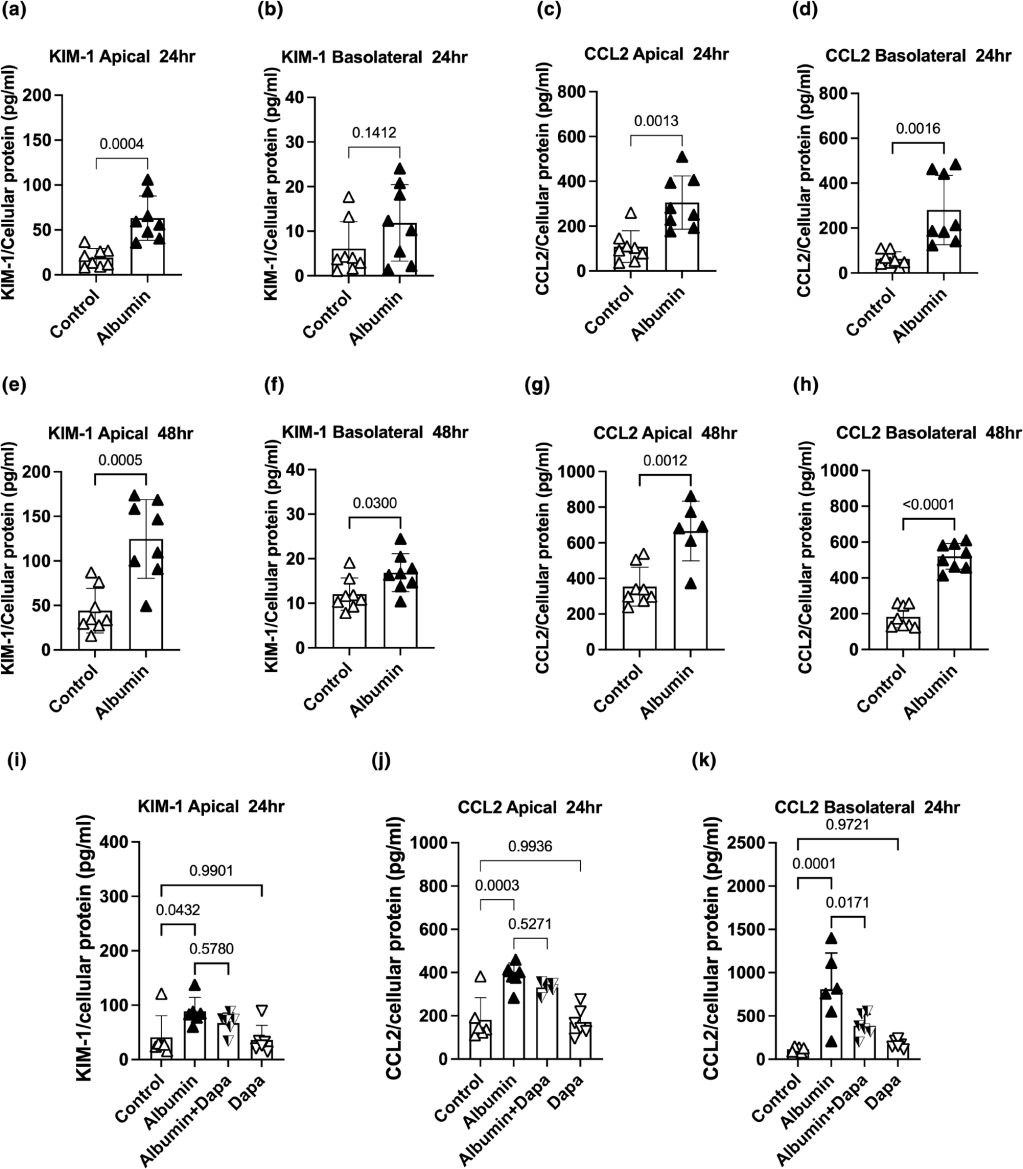
Albumin induces tubular secretion of KIM‐1 and CCL2, and SGLT2 inhibition attenuates albumin‐driven inflammatory signaling. Effects of albumin exposure on tubular injury markers and inflammatory chemokine secretion in human proximal tubular epithelial cells (RPTEC/TERT1). (a–d) Apical and basolateral secretion of KIM‐1 and CCL2 following 24 h exposure to albumin. (e–h) Apical and basolateral secretion of KIM‐1 and CCL2 following 48 h exposure to albumin. (i) Apical KIM‐1 secretion following 24 h treatment with albumin ± dapagliflozin (Dapa). (j, k) Apical and basolateral CCL2 secretion following 24 h treatment with albumin ± dapagliflozin. Data are expressed as protein concentration normalized to cellular protein content and presented as mean ± SD. Statistical comparisons between two groups (a‐h) were performed using unpaired Student's t‐test, multi‐group comparisons (i‐k) used one‐way ANOVA with Tukey's post hoc test. A *p*‐value < 0.05 was considered statistically significant.

To determine whether albumin stimulates tubular injury and inflammatory signaling, human proximal tubular epithelial cells (RPTEC/TERT1) were exposed to albumin, and apical and basolateral secretions of KIM‐1 and CCL2 were quantified at 24 and 48 h (Figure 4).

At 24 h, albumin exposure significantly increased apical KIM‐1 secretion compared with control conditions (Figure 4a, *p* = 0.0004), whereas basolateral KIM‐1 showed no significant increase at this time point (Figure 4b, *p* = 0.1412). In contrast, CCL2 secretion was robustly induced by albumin at 24 h, with significant increases observed in both the apical (Figure 4c, *p* = 0.0013) and basolateral (Figure 4d, *p* = 0.0016) compartments, indicating early activation of inflammatory signaling.

By 48 h, albumin‐induced tubular stress was further amplified. Apical KIM‐1 secretion remained significantly elevated (Figure 4e, *p* = 0.0005), and basolateral KIM‐1 was now significantly increased (Figure 4f, *p* = 0.0300), consistent with progressive epithelial injury. Similarly, CCL2 secretion continued to rise, with marked increases in both the apical (Figure 4g, *p* = 0.0012) and basolateral (Figure 4h, *p* <0.0001) compartments.

Together, these findings demonstrate that albumin induces tubular injury and CCL2 release in proximal tubular epithelial cells. We next examined whether pharmacologic modulation of proximal tubular transport, via SGLT2 inhibition, alters albumin‐induced inflammatory signaling. Cells were treated with dapagliflozin (Dapa) in the presence or absence of albumin for 24 h. Dapa did not significantly reduce albumin‐induced apical KIM‐1 secretion (Figure 4i). In contrast, Dapa significantly attenuated albumin‐induced CCL2 secretion in the basolateral compartment (Figure 4k, *p* = 0.0171), whereas it had no effect on apical CCL2 (Figure 4j).

Collectively, these findings demonstrate that in vitro albumin promotes CCL2 secretion and tubular injury in human proximal tubule cells. Further, Dapa attenuated CCL2 release across the basolateral membrane. Given that basolateral CCL2 would be exposed to the renal interstitium and peritubular capillaries and therefore potentially play a role in renal macrophage recruitment, we next examined whether SGLT2 inhibition modifies blood pressure and renal inflammation in response to HS intake in male Dahl SS rats.

### 3.5 SGLT2 inhibition attenuates the early response to salt‐induced hypertension

**FIGURE 5:**
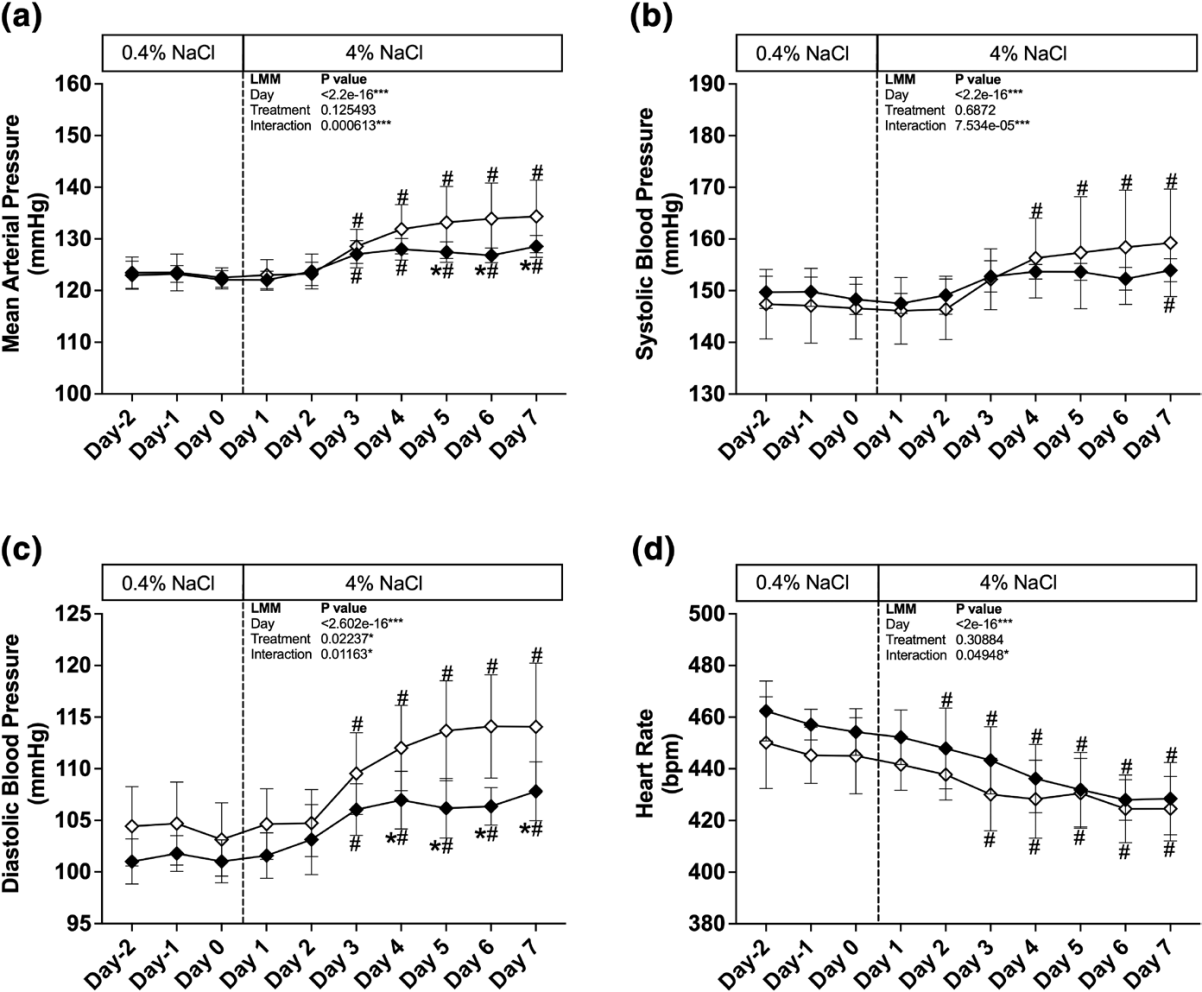
SGLT2 inhibition attenuates salt‐induced hypertension in male Dahl salt‐sensitive rats. Rats received either vehicle (white circles) or dapagliflozin (DAPA; black circles) mixed in Nutella throughout the study. (a) Mean arterial pressure (MAP), (b) systolic blood pressure (SBP), (c) diastolic blood pressure (DBP), and (d) heart rate (HR) were measured by telemetry during baseline control salt diet and following transition to a high salt diet. The dashed vertical line denotes initiation of the high‐salt diet (day 0). Data are presented as mean ± SD, *n* = 5,6/group. Statistical analysis was performed using a linear mixed‐effects model with day and treatment as fixed effects and animal as a random effect; corresponding *p* values for main effects and interactions are shown within each panel. Tukey's post hoc test was used for multiple comparisons. *p < 0.05 vehicle versus Dapa. #*p* < 0.05 versus baseline.

To determine whether SGLT2 inhibition modifies the early blood pressure response to HS intake, male SS rats were treated with Dapa or vehicle over 7 days of HS and blood pressure was monitored by radiotelemetry (Figure 5). MAP was significantly lower in HS‐Dapa rats relative to HS‐vehicle rats from day 5. Comparable results were obtained with DBP (Figure 5c), whereas SBP (Figure 5b) and HR (Figure 5d) did not differ between groups.

Given that Dapa attenuated the MAP response to HS, we next examined whether SGLT2 inhibition reduced renal injury and inflammation in HS fed rats.

### 3.6 Dapagliflozin increases diuresis and natriuresis without attenuating proteinuria or CCL2 excretion during high‐salt intake

**FIGURE 6:**
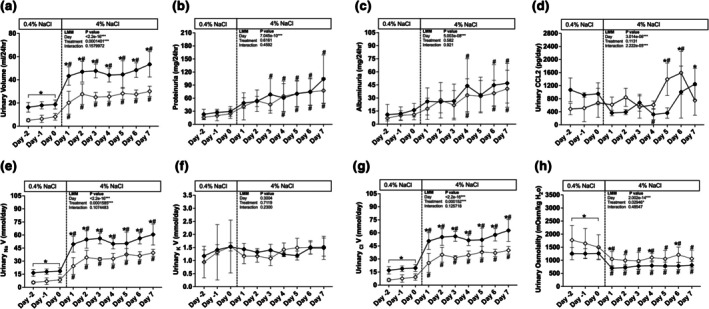
Effect of SGLT2 inhibition on urinary excretion, proteinuria, and inflammatory markers during high‐salt intake. Rats received vehicle (white circles) or dapagliflozin (DAPA; black circles) mixed in Nutella throughout the study. (a) Urinary volume, (b) proteinuria, (c) albuminuria, (d) urinary CCL2 excretion, (e) urinary sodium excretion, (f) urinary potassium excretion, (g) urinary chloride excretion, and (h) urinary osmolality were measured daily during baseline control salt diet and following transition to a high salt diet. The dashed vertical line denotes initiation of the high‐salt diet (day 0). Data are presented as mean ± SD, *n* = 5,6/group. Statistical analysis was performed using a linear mixed‐effects model with day and treatment as fixed effects and animal as a random effect, with Tukey's post hoc test for multiple comparisons. Corresponding *p* values for main effects and interactions are shown within each panel. *p < 0.05 vehicle versus Dapa. #*p* < 0.05 versus baseline.

Urine volume, protein, albumin, CCL2, and electrolyte excretion were assessed in HS‐vehicle and HS‐Dapa rats (Figure 6). Urine volume was significantly higher in HS‐Dapa rats than in HS‐vehicle rats throughout the dietary challenge, including during the baseline period during which both groups were fed a CS diet (Figure 6a). This was paralleled by increased urinary sodium and chloride and reduced urine osmolality (Figure 6e,g,h, respectively). As with urinary volume, urinary sodium was also higher in Dapa rats than in vehicle control rats during the baseline period. Urinary potassium did not differ between the groups (Figure 6f).

Neither proteinuria nor albuminuria differed between HS‐vehicle and HS‐Dapa rats (Figure 6b,c). Urinary CCL2 was not lower in HS‐Dapa rats than in HS‐vehicle rats (Figure 6d). A linear regression between average MAP and average urinary albumin excretion across the 7 days of HS revealed no significant correlation (*R* ^2^ = 0.34, *p* = 0.062).

Together, these data demonstrate that SGLT2 inhibition effectively increases diuresis and natriuresis during early salt loading, yet fails to attenuate proteinuria, albuminuria, or urinary CCL2. We next examined whether SGLT2 inhibition affected renal immune cell accumulation during early high‐salt intake.

### 3.7 Flow cytometric analysis confirms that Dapa does not alter renal immune cell composition during early high‐salt exposure

**FIGURE 7:**
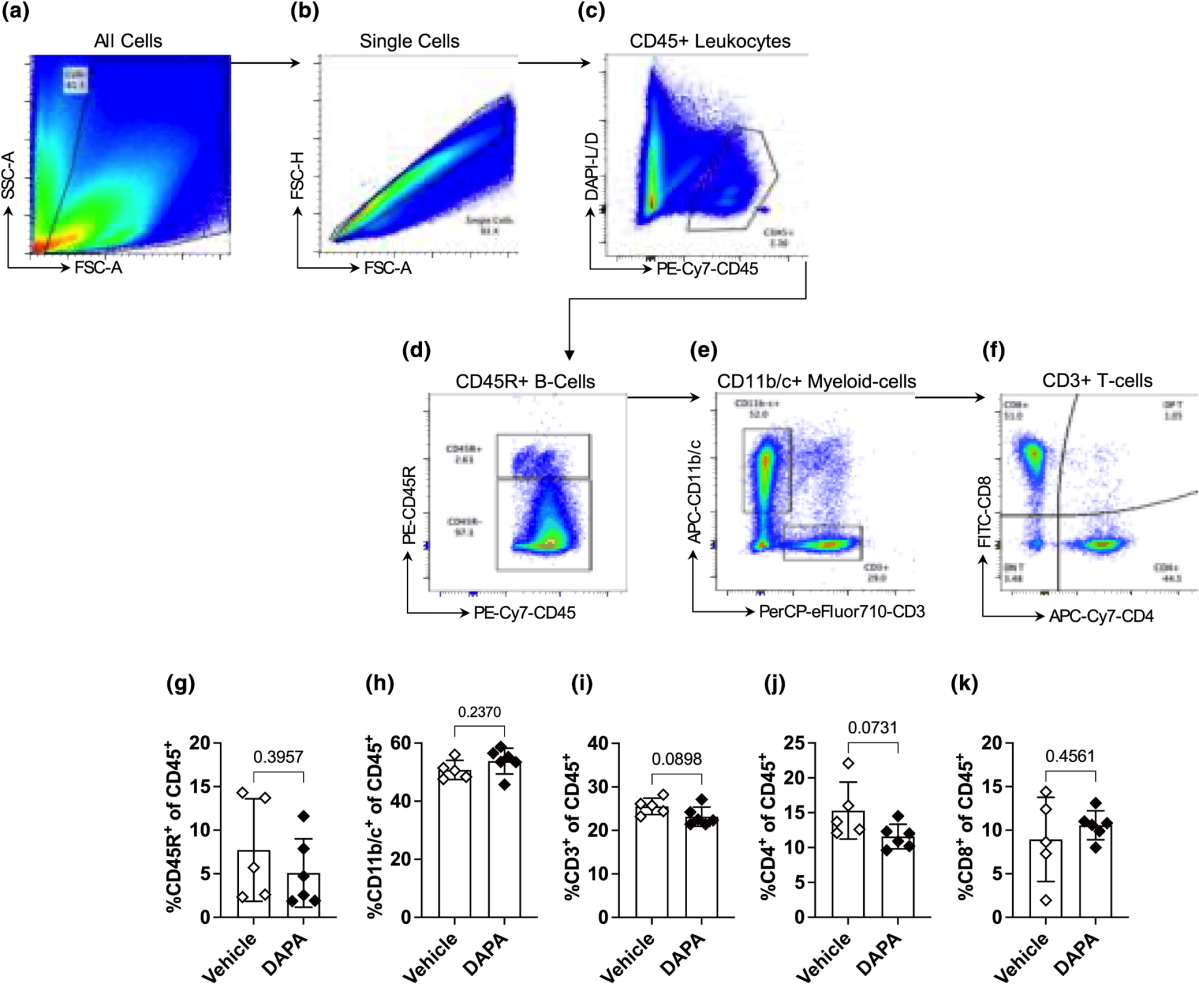
Flow cytometric analysis of renal immune cell populations following dapagliflozin treatment. Representative gating strategy showing (a) total cells, (b) single cells, and (c) live CD45^+^ leukocytes. Within the CD45^+^ population, identification of (d) CD45R^+^ B cells, (e) CD11b/c^+^ myeloid cells, and (f) CD3^+^ T cells with subsequent CD4^+^ and CD8^+^ subsets. Quantification of (g) CD45R^+^ B cells, (h) CD11b/c^+^ myeloid cells, (i) CD3^+^ T cells, (j) CD4^+^ T cells, and (k) CD8^+^ T cells expressed as a percentage of total CD45^+^ leukocytes. Data are presented as mean ± SD, *n* = 5,6/group. Statistical comparisons were performed using unpaired Student's *T*‐tests. A *p*‐value of < 0.05 was considered statistically significant.

To determine whether Dapa alters renal immune cell composition during early HS intake, flow cytometry was performed on single‐cell suspensions prepared from kidneys after 7 days of HS diet (Figure 7). Kidney single‐cell suspensions were gated sequentially to identify live, single CD45^+^ leukocytes, followed by separation of B cells (CD45R^+^), myeloid cells (CD11b/c^+^), and T‐cell subsets (CD3^+^, CD4^+^, and CD8^+^) (Figure 7a–f). Quantitative analysis showed no significant difference in the frequency of total CD45R^+^ B cells between HS‐vehicle and HS‐Dapa rats (Figure 7g, *p* = 0.3957). Similarly, the proportions of CD11b/c^+^myeloid cells were unchanged by Dapa treatment (Figure 7h, *p* = 0.2370). Analysis of lymphocyte subsets further revealed no effect of Dapa on renal T‐cell populations, including total CD3^+^ T cells, CD4^+^ helper T cells, or CD8^+^ cytotoxic T cells (Figure 7i–k, all *p* > 0.05). Together, these findings indicate that SGLT2 inhibition does not alter the overall composition of renal leukocyte populations during the early phase of salt‐sensitive hypertension. To further address whether proportional data may mask changes in absolute cell numbers, absolute immune cell counts per gram of kidney tissue were calculated. Absolute numbers of CD11b/c + myeloid cells (Vehicle: 28,783,200 ± 19,505,694 vs. DAPA: 20,791,867 ± 11,122,795 cells/g), CD3+ T cells (15,284,133 ± 10,691,361 vs. 10,394,244 ± 6,093,842 cells/g), CD4+ T cells (10,550,000 ± 9,611,379 vs. 5,373,367 ± 3,505,798 cells/g), CD8+ T cells (3,943,493 ± 2,508,853 vs. 4,578,158 ± 2,439,496 cells/g), and CD45R+ B cells (6,552,840 ± 7,057,957 vs. 2,905,317 ± 3,244,014 cells/g) did not differ significantly between groups (all *p* > 0.05), confirming that dapagliflozin did not alter absolute renal immune cell numbers during early high‐salt exposure.

### 3.8 Dapagliflozin does not alter renal macrophage or T‐cell accumulation during the HS diet

**FIGURE 8:**
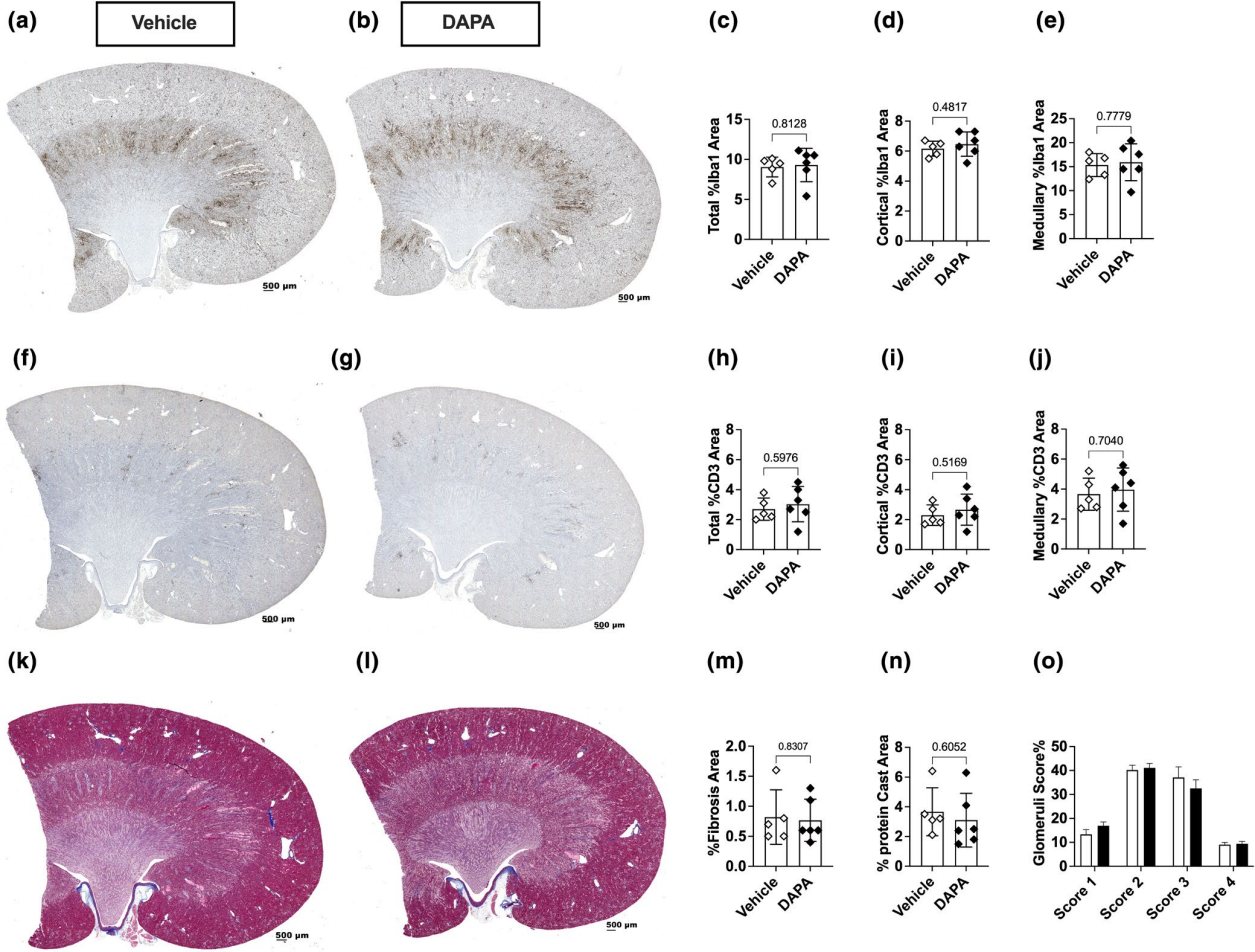
Immune cell infiltration and renal histological injury are not attenuated by dapagliflozin during early high‐salt exposure. Representative immunohistochemical staining for Iba1‐positive macrophages in (a) vehicle‐treated and (b) Dapa‐treated kidneys with corresponding quantification of (c) total, (d) cortical, and (e) medullary Iba1‐positive area. (f) Representative CD3 immunostaining in vehicle‐treated and (g) Dapa‐treated kidneys with quantification of (h) total, (i) cortical, and (j) medullary CD3‐positive area. Representative Masson's trichrome‐stained whole kidney sections from (k) vehicle‐treated and (l) Dapa‐treated rats are shown. Quantification of renal (m) fibrosis, (n) cortical protein cast area, and (o) glomerular injury score distribution is presented as a percentage of the total renal area. Data are presented as mean ± SD, *n* = 5,6/group. Statistical comparisons were performed using unpaired Student's *t*‐tests. A *p‐*value of < 0.05 was considered statistically significant.

To complement the flow cytometry findings and assess the distribution of immune cells within the kidney, immunohistochemical analyses were performed on whole kidney sections from HS‐vehicle and HS‐Dapa rats (Figure 8). Representative Iba1‐stained sections revealed a similar pattern of macrophage distribution between groups (Figure 8a,b). Quantification demonstrated no significant effect of Dapa on total Iba1^+^ area (Figure 8c, *p* = 0.8128), or on cortical and medullary Iba1^+^ staining (Figure 8d,e, *p* = 0.4817 and 0.7779, respectively). Consistent with the flow cytometry data, CD3 immunostaining revealed no significant differences in total, cortical, or medullary CD3^+^ T‐cell area between HS‐vehicle and HS‐Dapa rats (Figure 8f–j, *p* = 0.5976, 0.5169, and 0.7040, respectively).

Assessment of structural injury further demonstrated no differences in fibrosis, tubular protein cast area, or glomerular injury scores between groups (Figure 8k–o), indicating that Dapa treatment does not significantly alter renal immune accumulation or injury at this early stage of HS exposure.

## 4 DISCUSSION

Salt‐sensitive hypertension is closely linked to early renal injury and inflammation. However, the sequence of initiating events and the mechanisms coupling tubular injury to inflammation remain incompletely understood (Evans et al., 2017; Mattson, 2014). The present study provides several insights. First, we demonstrated that albuminuria is an early event that precedes differences in blood pressure between HS and CS fed SS rats. Second, albuminuria was accompanied by increased urinary CCL2 and renal macrophage accumulation, suggesting a potential role for albuminuria in initiating the early innate immune response. Albuminuria and macrophage accumulation occurred prior to differences in fibrosis, glomerular injury, or T‐cell accumulation in HS and CS fed rats. Third, using an immortalized human proximal tubule cell line, we showed that apical albumin can stimulate CCL2 secretion from tubular epithelial cells, an effect that can be prevented by SGLT2 inhibition with dapagliflozin. Finally, although dapagliflozin attenuated the MAP response to a HS diet in SS rats, it did not reduce albuminuria or renal inflammation over our short experimental time course, highlighting a dissociation between the initiation of hypertension and early inflammatory signaling. These studies were performed exclusively in male rats, since females are relatively protected from the hypertensive effects of a high‐salt diet (Dayton et al., 2016). Based on these data, we predict that urinary cytokine excretion and renal macrophage infiltration would be blunted in female SS; however, further studies to examine this are warranted.

The demonstration that urinary albumin was higher in HS fed SS than CS fed SS, prior to differences in blood pressure and glomerular injury between the groups, suggests that albuminuria is not simply a consequence of hypertension‐induced glomerular damage but may instead represent an initiating event in salt‐sensitive renal injury. Consistent with this, Poyan Mehr et al. demonstrated that albuminuria is elevated in SS rats as early as 4 weeks of age on a low‐sodium diet, independent of salt loading or blood pressure elevation, suggesting an inherent susceptibility to albumin leak in the SS kidney that is further amplified by high‐salt intake (Poyan Mehr et al., 2003). Proteinuria and albuminuria are increasingly recognized as active mediators of tubulointerstitial injury rather than a biomarker (Tang et al., 2003; Zoja et al., 1998). Filtered albumin is reabsorbed by proximal tubule cells through receptor‐mediated endocytosis, where it can activate inflammatory signaling cascades, oxidative stress, and cytokine production (Liu et al., 2015; Wang et al., 1999). In the current study, albuminuria was paralleled by elevated urinary CCL2 levels and increased renal macrophage accumulation, as assessed by Iba1 immunostaining. Notably, T‐cell infiltration was not increased at this early time‐point. Based on these data, we propose that albumin‐driven signaling may activate innate immune pathways during the early phase of salt‐sensitive hypertension. CCL2 is a potent chemoattractant for monocytes and plays a central role in inflammation (Deshmane et al., 2009). Our correlation analysis in combination with our in vitro studies suggests that CCL2 produced by the proximal tubule in response to albuminuria could play a role in the renal accumulation of macrophages. Previous studies in SS rats have demonstrated that after 3 days on an 8% NaCl diet, there is increased proximal tubular albumin reabsorption, a compensatory response to increased filtration. By day 14 of 8% NaCl, the proximal tubules were “flooded” with albumin, which was associated with tubular injury (Endres et al., 2017). The current study adds to this by demonstrating higher albuminuria in HS fed SS rats after only 1 day on a 4% NaCl diet. It should also be noted that systemic blood pressure may not fully reflect intraglomerular pressure. Even in the absence of detectable differences in mean arterial pressure between groups, early afferent arteriolar dilation or altered autoregulatory responses to high salt could result in increased glomerular capillary pressure, which may itself contribute to the early albuminuria observed. Direct measurement of glomerular pressure would be required to fully resolve this question and represents an important avenue for future investigation.

Despite the early increases in urinary cytokines and renal macrophage accumulation in HS fed SS rats, glomeruli injury scores and fibrosis were not different from CS fed SS rats. This finding is consistent with the notion that innate inflammation precedes most structural damage. These data emphasize the importance of targeting early inflammatory signaling before chronic injury.

Interestingly, despite the ability of dapagliflozin to reduce CCL2 release from proximal tubule cells in vitro, it did not mitigate proteinuria, albuminuria, renal macrophage accumulation, or renal injury during the early phase of salt‐sensitive hypertension in SS rats. There are several possible explanations for this. First, while the short duration of this study was by design to characterize the early phase of salt‐sensitive hypertension, it may have been insufficient to observe any potential anti‐inflammatory effects of dapagliflozin. Second, systemic and hemodynamic effects of dapagliflozin may dominate early outcomes, whereas anti‐inflammatory benefits may emerge only with longer treatment or more advanced disease. Thirdly, dapagliflozin treatment may have paradoxically increased salt intake in our rats, as demonstrated by persistently higher urine sodium levels prior to the high salt diet. Thus, the anti‐inflammatory impacts of SGLT2 may be confounded by the pro‐inflammatory impact of higher sodium intake (Kirabo, 2017). Further delineation of these impacts would be a fruitful avenue for future study. Additionally, renal mRNA expression of pro‐inflammatory cytokines (TGF‐β, TNF‐α, IL‐1β) and tubular injury markers (lipocalin‐2/NGAL, KIM‐1) was not assessed in the current study. Transcript‐level measurements may be more sensitive than urinary protein excretion for detecting early inflammatory responses and could further clarify whether a longer duration of dapagliflozin treatment would unmask additional anti‐inflammatory effects. Future studies should address this gap.

In summary, we have demonstrated that albuminuria is an early event in the development of salt‐sensitive hypertension in Dahl salt‐sensitive rats. Urinary albumin increased within 1 day of high salt intake, preceding measurable differences in mean arterial pressure and occurring in parallel with elevated urinary CCL2. After 7 days of high salt, urinary CCL2 correlated positively with renal macrophage accumulation. These inflammatory changes were observed in the absence of fibrosis, glomerular injury, or T‐cell infiltration, consistent with activation of innate immune pathways during the primary phase of salt‐sensitive renal injury. Using a polarized human proximal tubule cell model, we further show that luminal albumin stimulates CCL2 secretion from tubular epithelial cells and that this response is prevented by SGLT2 inhibition with dapagliflozin. It is important to note that these in vitro studies were performed in human cells, and species‐specific differences in chemokine signaling or SGLT2‐dependent pathways may influence the extent to which these responses are recapitulated in vivo. Despite these anti‐inflammatory effects in vitro, dapagliflozin treatment in vivo attenuated the blood pressure response to high salt without reducing albuminuria or early renal inflammation.

## AUTHOR CONTRIBUTIONS

**Rawan N. Almutlaq:** Conceptualization; data curation; formal analysis; investigation; methodology. **Yotesawee Srisomboon:** Investigation; methodology. **Sridhatri Guntipally:** Investigation. **Andrew N. Hakeem:** Investigation. **Amanda C. Veiga:** Investigation. **Jaryd Ross:** Investigation. **Babatunde S. Anidu:** Investigation. **Alex Dayton:** Conceptualization; formal analysis; investigation; methodology. **Scott M. O'Grady:** Conceptualization; formal analysis; investigation; methodology. **Louise C. Evans:** Conceptualization; data curation; formal analysis; funding acquisition; investigation; methodology; supervision.

## FUNDING INFORMATION

This work is funded by the National Heart, Lung, and Blood Institute Grant R01HL152166 to LCE.

## CONFLICT OF INTEREST STATEMENT

The authors declare no conflicts of interest.

## ETHICS STATEMENT

All animal procedures were approved by the Institutional Animal Care and Use Committee (IACUC) at the University of Minnesota and conducted in accordance with the NIH Guide for the Care and Use of Laboratory Animals.

## Data Availability

Data will be made available upon reasonable request.
