# Correction to “Inhibition of SGLT2 reduces blood pressure in the early phase of salt‐sensitive hypertension in male Dahl‐SS rats independently of changes in renal inflammation”

**DOI:** 10.14814/phy2.71034

**Published:** 2026-07-31

Almutlaq, R. N. , Srisomboon, Y. , Guntipally, S. , Hakeem, A. N. , Veiga, A. C. , Ross, J. , Anidu, B. S. , Dayton, A. , O'Grady, S. M. , & Evans, L. C. (2026). Inhibition of SGLT2 reduces blood pressure in the early phase of salt‐sensitive hypertension in male Dahl‐SS rats independently of changes in renal inflammation. Physiological Reports, 14, e70998. 10.14814/phy2.70998.42397179 PMC13330592

In Figures 3i, 3j, 3n, and 3o, the *x*‐axis units of (mg/ml) were incorrect. These should have read “mg/24 hr.”

The experiments were correctly described in the text and the corrected figure is provided below:

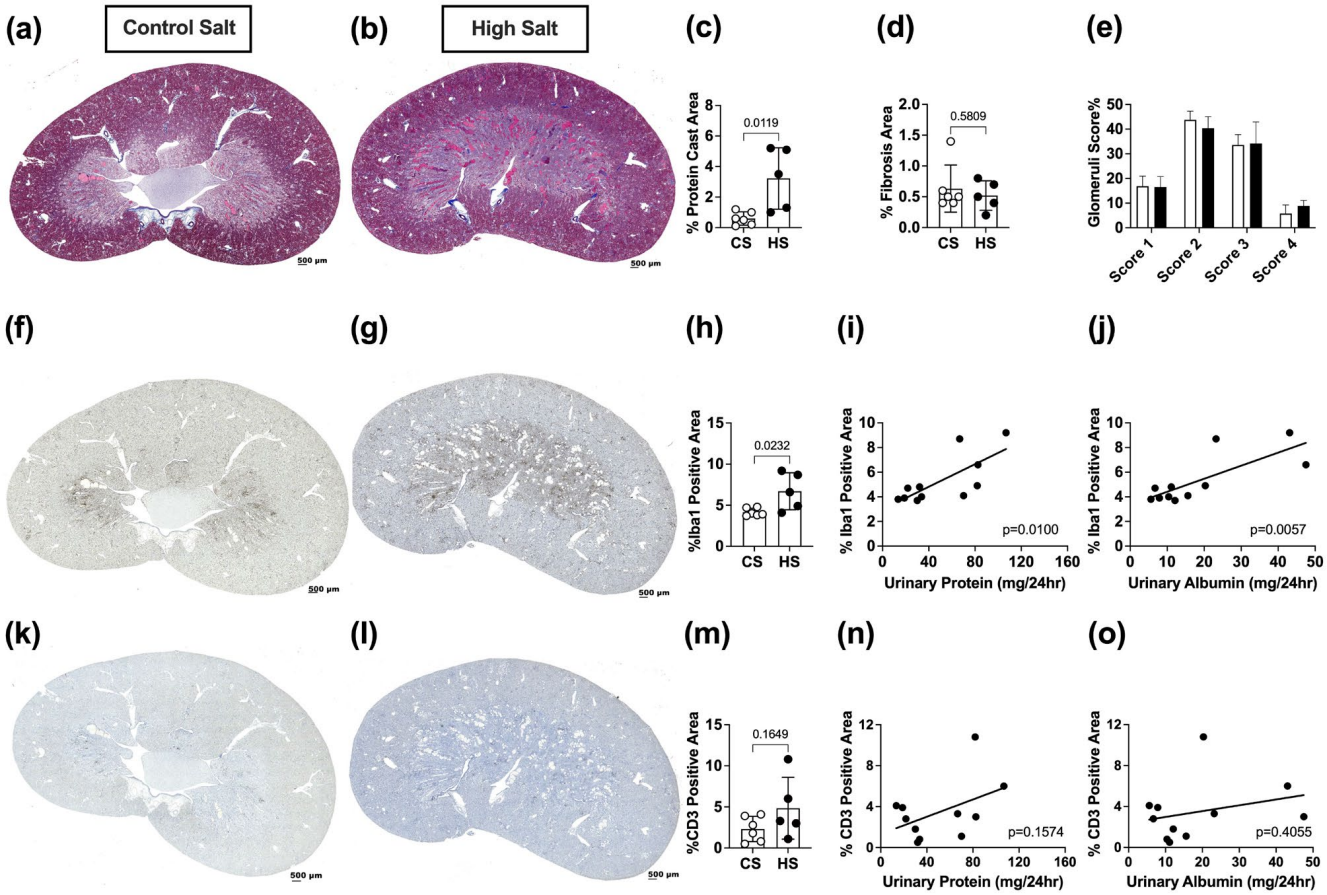

We apologize for this error, which does not alter the scientific conclusions of the study.

